# The Proapoptotic Effect of MB-653 Is Associated with the Modulation of Metastasis and Invasiveness-Related Signalling Pathways in Human Colorectal Cancer Cells

**DOI:** 10.3390/biom15010072

**Published:** 2025-01-06

**Authors:** Libor Sokoli, Peter Takáč, Mariana Budovská, Radka Michalková, Martin Kello, Natália Nosálová, Ľudmila Balážová, Šimon Salanci, Ján Mojžiš

**Affiliations:** 1 Department of Pharmacology and Toxicology, University of Veterinary Medicine and Pharmacy, Komenského 73, 041 81 Košice, Slovakia; liborsokoli@gmail.com; 2 Department of Pharmacology, Faculty of Medicine, Pavol Jozef Šafárik University, 040 01 Košice, Slovakia; radka.michalkova@upjs.sk (R.M.); martin.kello@upjs.sk (M.K.);; 3 Department of Organic Chemistry, Institute of Chemistry, Faculty of Science, Pavol Jozef Šafárik University, 040 01 Košice, Slovakia; 4 Small Animal Clinic, University of Veterinary Medicine and Pharmacy, Komenského 73, 041 81 Košice, Slovakia; natalia.nosalova@uvlf.sk; 5 Department of Pharmaceutical Technology, Pharmacognosy and Botany, University of Veterinary Medicine and Pharmacy, 041 81 Košice, Slovakia; ludmila.balazova@uvlf.sk

**Keywords:** indole phytoalexins, colorectal cancer, apoptosis, metastasis, EMT

## Abstract

Colorectal cancer is one of the most common cancers worldwide and has a high mortality rate. In this study, we investigated the cytotoxic, proapoptotic, and anti-invasive effects of the synthetic indole phytoalexin MB-653. The antiproliferative effect was determined using an MTT assay, showing IC_50_ values of 5.8 ± 0.3 μmol/L for HCT116 cells and 6.1 ± 2.1 μmol/L for Caco2 cells. Flow cytometry and Western blot analysis were employed to investigate the molecular mechanisms underlying cytotoxicity, proapoptotic action, and anti-invasion effects. The proapoptotic activity was evidenced by the activation of caspases 3 and 7, mitochondrial dysfunction, and an increased number of apoptotic cells, confirmed by annexin V/PI and AO/PI staining. Additionally, MB-653 induces dose-dependent G2/M phase cell cycle arrest, the cause of which could be cyclin B1/CDC2 complex dysfunction and/or a decrease in α-tubulin protein expression. Another important observation was that MB-653 modulated several signalling pathways associated with various cellular activities, including survival, proliferation, tumour invasiveness, metastasis, and epithelial–mesenchymal transition (EMT). We further demonstrated its safety for topical and parenteral application. To sum up, our results indicate the real potential of MB-653 in treating colorectal cancer.

## 1. Introduction

Colorectal cancer (CRC) has a high mortality rate, and early detection is crucial for improving prognosis and reducing mortality associated with CRC [1]. The primary risk factor for CRC is age, with both incidence and mortality rates increasing with age. For instance, the incidence and mortality rates of CRC rise significantly from 6 and 1 per 100,000 for individuals aged 30–34 years to 228 and 105 per 100,000 for those aged 80–84 years (colon and rectum SEER incidence and U.S. mortality rates by age at diagnosis, 2014–2018).

The lifetime risk of developing CRC is similar in women and men, estimated at 4.1% and 4.4%, respectively. However, the increase in CRC incidence and mortality tends to occur later in women compared to men. Furthermore, the incidence of CRC can vary considerably based on family history and genetic risk factors, while mortality rates can also differ according to race/ethnicity and structural factors [2].

Some natural compounds found in plants, fungi, marine animals, and bacteria have been shown to inhibit the occurrence and development of various types of cancer, including CRC [3,4,5]. There is growing evidence that natural products possess anti-inflammatory, anti-ageing, antibacterial, and antiviral properties in vivo and in vitro and can suppress carcinogenesis, cancer progression, and the creation of micro- and macromolecules. They are readily accessible, cost-effective, structurally varied, abundant in biologically active molecules, and exhibit little side effects; hence, they are significant in the advancement of innovative anticancer pharmaceuticals and lead compounds [6].

Plants belonging to the *Brassicaceae* family, like broccoli, mustard, and beet, synthesize different indole- and sulphur-based compounds in response to pathogen attacks. These substances, known as phytoalexins, are believed to play a significant role in the plant’s defence system. Certain phytoalexins have demonstrated anticancer properties, suggesting that consuming a diet abundant in these vegetables could be beneficial for human health [2].

In recent decades, the number of evidence for the beneficial anticancer effects of phytoalexins has been increasing. The most studied indole phytoalexins in relation to cancer include camalexin and brassinin (and its isomers). Previous research has shown that camalexin, naturally occurring indole phytoalexin, inhibited the proliferation of human leukemia cells [7,8]. Brassinin, one of the first isolated cruciferous indole phytoalexin ever, has been tested on murine melanoma (B16) and leukemia (L1210) cell lines, demonstrating growth inhibition [9]. When combined with capsaicin, brassinin enhanced apoptotic and antimetastatic effects in human prostate carcinoma (PC-3) cells. Additionally, brassinin increased sensitivity to vincristine cytotoxicity in human glioblastoma astrocytoma (U-87 MG) cells [10]. Isobrassinin, a regioisomer of brassinin, exhibited antiproliferative effects on cervical carcinoma (HeLa), breast carcinoma (MCF-7), and epidermoid carcinoma (A431) cell lines [11].

Concerning the link between the indole phytoalexins and CRC research, previous studies in our laboratory have shown that 1-methoxybrassinin induces apoptosis in Caco2 cells and is associated with the upregulation of the proapoptotic *Bax* gene expression, downregulation of the anti-apoptotic *Bcl*-2 gene, activation of caspase-3 and -7, cleavage of Poly(ADP-ribose) polymerase (PARP), and reduction in intracellular glutathione (GSH) content [12]. Tischlerová et al., 2017 [13] describe compound K-453 {(±)-trans-1,2-dimethoxy-2′-(3,5-bis-trifluoromethylphenylamino)spiro{indoline-3,5′[4′,5′]dihydrotriazole} with activity IC_50_ = 32.22 ± 1.14 μmol/L in human colorectal HCT116 cells. K-453 exhibited an antiproliferative effect by induction of intrinsic apoptosis as well as modulation of several signalling pathways.

Although the number of evidence confirming the favourable effects of indole phytoalexins and their synthetic derivatives in relation to cancer prevention and/or therapy is constantly growing, still little is known about their potential to modulate key features of malignant processes such as invasion, metastasis, or EMT. This can also be said about one of the most dangerous cancers both in men and women: colorectal cancer. This fact prompted us to evaluate the proapoptotic, anti-invasive, and anti-metastatic potential of compound MB-653 (a synthetic derivative of natural indole phytoalexin spirobrassinin) in human colon cancer cell lines of two types.

## 2. Materials and Methods

### 2.1. Tested Compound

**Figure 1:**
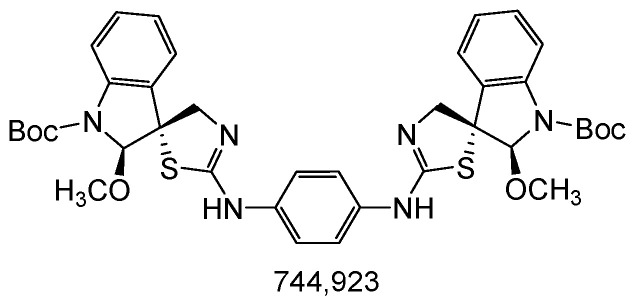
MB-653(trans-(±)-N,N’-bis [1-(tert-butoxycarbonyl)-2-methoxy-spiro{indoline-3,5′-[4′,5′]dihydrotriazol-2′-yl}]benzene-1,4-diamine).

trans-(±)-N,N’-bis [1-(tert-butoxycarbonyl)-2-methoxy-spiro{indoline-3,5′-[4′,5′]dihydrotriazol-2′-yl}]benzene-1,4-diamine (MB-653) is shown in Figure 1. The structure of the compound was confirmed using 1H and 13C NMR, IR spectroscopy, and mass spectrometry.

The compound was synthesized at the Faculty of Natural Sciences of P.J. Šafarik University, Košice (Mariana Budovska), and dissolved in DMSO. The final concentration of DMSO in the culture medium was kept below 0.2% and showed no cytotoxic effects.

### 2.2. Cell Cultures

The cell line HCT116 (human colorectal carcinoma, European Collection of Authenticated Cell Cultures, Salisbury, UK (ECACC)) was cultured in RPMI 1640 medium (Biosera, Kansas City, MO, USA), and Caco2 (human colorectal adenocarcinoma, American Type Culture Collection, Manassas, VA, USA (ATCC) cells were grown in a culture medium composed of high glucose Dulbecco’s Modified Eagle’s Medium (DMEM) supplemented with sodium pyruvate (GE Healthcare, Piscataway, NJ, USA). The growth medium was supplemented with a 10% fetal bovine serum, 1X HyClone™ Antibiotic/Antimycotic Solution (GE Healthcare, Little Chalfont, UK). The cells were incubated in a controlled environment with 5% CO_2_ in humidified air at 37 °C. Before each experiment, cell viability, assessed using trypan exclusion, consistently exceeded 95%. The MCF-10A (non-malignant breast epithelial cells, (ATCC)) cells were grown in a culture medium that included high-glucose Dulbecco’s Modified Eagle’s Medium F12 (DMEM F12; Biosera, Kansas City, MO, USA) with added antibiotic/antimycotic solution, insulin (final concentration of 10 µg/mL), EGF (20 ng/mL final concentration), hydrocortisone (final concentration of 0.5 µg/mL) (all from Merck, Darmstadt, Germany), and 10% fetal bovine serum. The cells were kept in a humidified environment with 5% CO_2_ at 37 °C. Before all experiments, cell viability exceeded 95%.

### 2.3. MTT Cell Proliferation/Viability Assay

The impact of synthetic indole phytoalexin (MB-653) on cancer cell line viability and proliferation was assessed using the 3-(4,5-dimethylthiazol-2-yl) 2,5-diphenyltetrazolium bromide (MTT) assay. Cells were plated at a density of 5 × 10^3^ cells per well in 96-well polystyrene microplates (SARSTEDT, Nümbrecht, Germany). After 24 h after seeding, various concentrations of the tested compounds (100, 50, 10, 5, and 1 μmol/L) were added. Following a 72 h incubation period, MTT solution (10 μL of 5 mg/mL, Sigma-Aldrich Chemie, Steinheim, Germany) was added to each well and incubated at 37 °C for 4 h to allow formazan formation. Subsequently, 100 μL of 10% sodium dodecyl sulphate (SDS) was added to dissolve the formazan over an additional 12 h incubation period. The absorbance was then measured at 540 nm using the automated Cytation™ 3 Cell Imaging Multi-Mode Reader (Biotek, Winooski, VT, USA). Three independent experiments were conducted for each test. The results obtained from the MTT assay were utilized to determine the half-maximal inhibitory concentration (IC_50_) of each tested compound.

### 2.4. Cell Cycle Analysis

To analyze the cell cycle using flow cytometry, both floating and adherent cells were collected together at 24, 48, and 72 h post-treatment. The collected cells were washed with cold PBS, fixed in cold 70% ethanol, and stored at −20 °C overnight. Before analysis, the cells were washed twice with PBS and then resuspended in a staining solution containing 0.1% Triton X-100, 0.5 mg/mL ribonuclease A, and 0.025 mg/mL propidium iodide (PI). The cell suspension was incubated in the dark at room temperature for 30 min and then analyzed using a FACSCalibur flow cytometer (Becton Dickinson, San Jose, CA, USA).

### 2.5. Flow Cytometric Analyses

HCT116 cells (1 × 10^6^) and Caco2 cells (1 × 10^6^) were treated with 10 μmol/L concentration of MB-653 for 24, 48, and 72 h. Both floating and adherent cells were harvested, washed with PBS, and divided for specific analyses. Fluorescence signals were detected after 15 min of incubation at room temperature in the dark using a BD FACSCalibur flow cytometer (Becton Dickinson, San Jose, CA, USA). A minimum of 10,000 events were analyzed per experiment.

#### 2.5.1. Detection of Mitochondrial Membrane Potential Changes

Mitochondrial membrane potential (MMP) was assessed by measuring the retention of the red-orange positively charged dye tetramethylrhodamine ethyl ester (TMRE; Molecular Probes, Eugene, OR, USA). The cells were treated with TMRE and incubated for 30 min at room temperature in the dark. Fluorescence emitted by TMRE (excitation/emission wavelength of 549/574 nm) was detected using the FL-2 (585/42) channel. The fluorescence data were analyzed using FlowJo software v.10 (BD Biosciences, San Jose, CA, USA).

#### 2.5.2. Apoptosis Detection via Externalized Phosphatidylserine

The plasma membrane changes characteristic of apoptosis were assessed using An-nexin V-Alexa Fluor^®^ 647 and propidium iodide (PI) double staining following the manufacturer’s instructions. Adherent and floating cells (1 × 105) were collected at 24, 48, and 72 h post-treatment. The cells were first stained with Annexin V–Alexa Fluor^®^ 647 (Thermo Scientific, Rockford, IL, USA) in binding buffer for 15 min, washed, and then stained with PI for 5 min. Subsequently, flow cytometric analysis was performed using a BD FACSCalibur flow cytometer. Based on the staining patterns, three cell populations were identified: viable cells (Annexin V–Alexa Fluor^®^ 647 negative and PI negative), apoptotic cells (Annexin V–Alexa Fluor^®^ 647 positive and PI negative), and late apoptotic/necrotic cells (Annexin V–Alexa Fluor^®^ 647 positive and PI positive, or Annexin V–Alexa Fluor^®^ 647 negative and PI positive).

The externalization of phosphatidylserine, an indicator of early and late apoptosis, was examined through Annexin V and PI staining. Cells were resuspended in PBS and stained with Annexin V–Alexa Fluor^®^ 647 (Thermo Scientific, Rockford, IL, USA) for 15 min in the dark, followed by washing in PBS. Then, 1 µL of PI (0.025 mg/mL) (Sigma Aldrich, St. Louis, MO, USA) was added to the samples. Flow cytometry analysis was performed using FL-2 (585/42) vs. FL-4 (661/16) channels, with a minimum of 10,000 events analyzed per sample. All experiments were conducted in triplicate, and FlowJo software v.10 (BD Biosciences, San Jose, CA, USA) was used for data analysis.

#### 2.5.3. Detection of Caspase 3/7 Activity

Caspase-3/7 activity was assessed following the manufacturer’s protocol. To investigate whether MB-653 activated effector caspases-3 and -7, we utilized a DEVD (Asp-Glu-Val-Asp) peptide substrate linked to a DNA binding dye. Upon apoptosis induction, active caspases-3/7 cleaved the DEVD peptide, leading to dye release, translocation to the nucleus, dye binding to DNA, and subsequent fluorescence emission. CellEvent™ Caspase-3/7 Green Detection Reagent was added to each flow cytometry tube and incubated for 30 min at 37 °C in the dark. Five minutes before measurement, cells were stained with SYTOX™ AADvanced™ Dead Cell Stain. Fluorescence signals were detected using the FL-1 (530/30) vs. FL-4 (661/16) channels on a flow cytometer. The acquired data were analyzed using FlowJo software v.10 (BD Biosciences, San Jose, CA, USA).

### 2.6. AO/PI Staining

Apoptosis occurrence was assessed using AO/PI staining and fluorescence microscopy. Acridine orange (AO) and propidium iodide (PI) are fluorescent dyes that bind to nucleic acids. AO can penetrate cells with intact membranes, while PI enters cells with compromised membranes. This staining method allows for the classification of cells into living (green fluorescence), apoptotic (yellow/orange fluorescence), and dead (red fluorescence) categories.

HCT116 and Caco2 cells were seeded in 6-well plates (1 × 10^5^ cells/well) and treated with compound MB-653 for 24, 48, and 72 h. After treatment, cells were washed with PBS, fixed in 4% paraformaldehyde for 20 min, and then washed again with PBS. A staining solution containing acridine orange (AO) and propidium iodide (PI) at a final concentration of 10 µg/mL each was prepared using reagents from Sigma Aldrich (St. Louis, MO, USA). The staining solution was added to the wells, and cells were incubated in the dark at room temperature for 1 h.

After incubation, cells were washed with PBS, and the plates were dried. The stained cells were then imaged and analyzed using an automated Cytation™ 3 Cell Imaging Multi-Mode Reader (Biotek, Winooski, VT, USA) equipped with excitation filters of 360/40 nm and 485/20 nm and emitting filters of 460/40 nm, 528/20 nm, and 620/40 nm.

### 2.7. Western Blot Analysis

Western blot analysis was conducted to assess protein levels. HCT116 and Caco2 cells were cultured in Petri dishes at a density of 1 × 10^6^ cells/dish and treated with MB-653 for 24, 48, and 72 h. Following treatment, protein lysates were prepared using Laemmle lysis buffer containing glycerol, 20% SDS, 1 M Tris/HCl (pH = 8.6), deionized H_2_O, and protease and phosphatase inhibitors, followed by sonication. Protein concentrations were determined using the Pierce^®^ BCA protein assay kit (Bradenton, FL, USA) and a Cytation™ 3 Cell Imaging Multi-Mode Reader at a wavelength of 570 nm.

Samples (40 ng) were loaded onto a 10% SDS-PAA gel and separated by electrophoresis at 100 V for 3 h. Proteins from the gel were then transferred to a PVDF membrane using the iBlotTM dry blotting system. The membranes were blocked with 5% dry non-fat milk or 5% BSA in TBS-Tween (pH = 7.4) for 1 h at room temperature to reduce nonspecific binding. Subsequently, membranes were incubated with primary antibodies (refer to Table 1) overnight at 4 °C, followed by incubation with a horseradish peroxidase-conjugated secondary antibody.

The expression of specific proteins was visualized using an ECL chemiluminescent substrate and detected with the MF-ChemiBIS 2.0 Imaging System and iBright™ FL1500 Imaging System. Densitometric analysis was performed using Image Studio Lite software (Version 5.2.5, LI-COR Biosciences, Lincoln, NE, USA) and iBright Analysis software (Version 5.2.5, Thermo Fisher Scientific, Cleveland, OH, USA).

### 2.8. In Ovo Methods

The laid fertile White Leghorn eggs from the certified hatchery Párovské Háje (Nitra, Slovakia) were cleaned and disinfected with 70% ethanol. The eggs were then incubated horizontally at 37.5 °C with a relative humidity of 60 ± 5% in a River System incubator (River, Italy). On the second embryonic day, three millilitres of albumin were removed using a sterile syringe and needle. The puncture site was then sealed with parafilm to prevent albumin leakage and contamination. Then, the eggs were returned to the incubator under the same controlled conditions. On embryonic day 8, a small hole was created in the upper portion of the eggshell using sterilized scissors to facilitate visualization of the chorioallantoic membrane (CAM). A sterilized silicone ring was then positioned on the CAM. The irritancy potential and angiogenesis were assessed using methods described in the following sections. The chick embryo is considered an experimental model. Approval of the animal protocol is not necessary, as it is exempt under the legislation regarding the protection of animals used for scientific purposes (2010/63/EU), as well as applicable laws in the United States.

#### 2.8.1. Irritancy Potential

The safety of the tested substances was evaluated using the Hen’s Egg Test on the CAM (HET-CAM) according to the ICCVAM-recommended protocol (NIH Publication No. 10-7553, 2010). Before application, the substances were diluted in sodium dodecyl sulphate (SDS) followed by 0.9% (*w/v*) NaCl to achieve a concentration of 10, 25, and 50 μM/mL. A volume of 30 µL of the prepared solution was applied to the silicone ring positioned on the CAM. Photographic documentation of the ring area was conducted using an Olympus SZ61 stereomicroscope (Tokyo, Japan) equipped with a PROMICRA 3.2 digital camera (Prague, Czech Republic) before application and at 30, 120, and 300 s post-application. The captured images were subsequently processed using QuickPHOTO MICRO software version 3.2 (Promicra, Prague, Czech Republic). The evaluation of irritancy was conducted by three independent scientists to minimize subjective errors, focusing on indicators such as lysis of vessels, hemorrhage, and coagulation. A numerical time-dependent score for these changes was assigned based on established criteria, as outlined in the accompanying Table 2 (ICCVAM, NIH Publication No. 10-7553, 2010).

#### 2.8.2. Angiogenesis

To assess angiogenesis, the same diluted substances used in the previous section (Section 2.8.1) were applied to the silicone ring positioned on the chorioallantoic membrane (CAM). The ring was photographed immediately before application and again after 72 h using the same camera and the software. The resulting images were evaluated for quantitative parameters, including total vessel area, total vessel length, mean vessel thickness, and the number of branching points. These measurements were conducted using IKOSA^®^ software (KOLAIDO GmbH, Altenrhein, Switzerland).

### 2.9. Statistical Analysis

The data are presented as the mean ± standard deviation (SD) from three separate experiments. Statistical analysis was performed using one-way analysis of variance (ANOVA) followed by Bonferroni’s test for multiple comparisons. For statistical evaluation, * indicates *p* < 0.05, ** indicates *p* < 0.01, and *** indicates *p* < 0.001 compared to the Control. All experiments were performed in three independent replicates.

## 3. Results

### 3.1. Effect of MB-653 on Cell Viability

The MTT colorimetric assay was used to test the effects of MB-653 on the proliferation of HCT116 and Caco2 cell lines. The data from this assay showed that the compound we tested had a concentration-dependent antiproliferative effect on both cell lines. IC_50_ values of 5.8 ± 0.3 μmol/L and 6.1 ± 2.1 μmol/L for the HCT116 and Caco2 cell lines, respectively, were obtained after 72 h from the MTT reduction assay, which measured the metabolic activity of the cells. The selectivity index against non-tumour cells was determined using MCF-10A cells, where the IC_50_ value was found to be 10.13 ± 6.2 (Table 3). The Cis-Pt assay was performed on HCT116, Caco2, and MCF-10A cells according to Nosalova et al. (2023) [14], and the results are shown in Table 3.

### 3.2. Cell Cycle Analysis and Cell Cycle–Related Proteins

**Figure 2:**
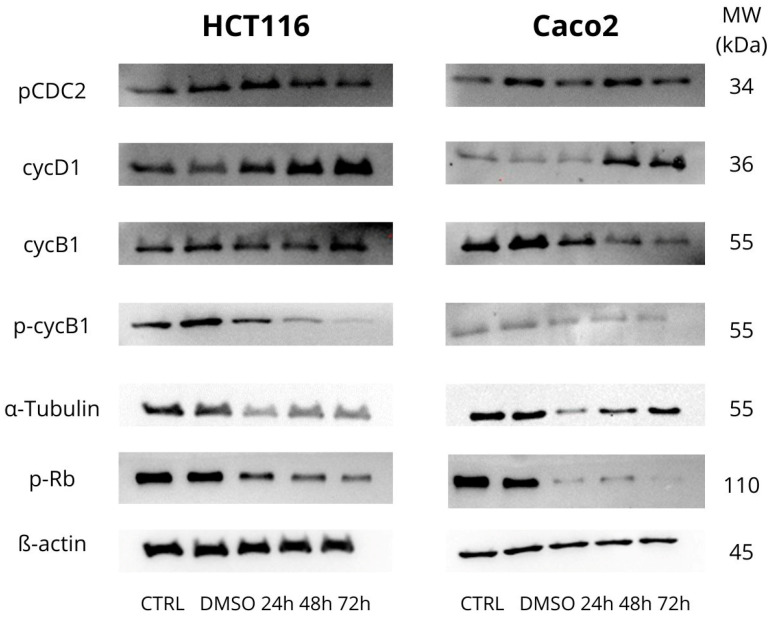
Western blot analysis of cell cycle-associated proteins affected by MB-653 in HCT116 and Caco2 cells after 24, 48, and 72 h incubation. Representative figure.

In examining the cell cycle, we focused on several cell cycle-associated proteins. The cell cycle was analyzed by flow cytometry, and protein levels were determined using Western blot analysis after incubation with MB-653 for 24, 48, and 72 h. The proteins assessed included Cyclin D1, Cyclin B1, tubulins, and pCDC2—Figure 2.

To evaluate the viability of colon cancer cells following MB-653 treatment, we performed flow cytometry. As shown in Table 4, MB-653 induced significant G2/M phase arrest in both cell lines after 24, 48, and 72 h of incubation, accompanied by changes in the number of cells in the G1 and S phases. The most pronounced effects were observed in Caco2 cells after 72 h and in HCT116 cells after 48 and 72 h of exposure (Supplement Figure S1).

**Figure 3:**
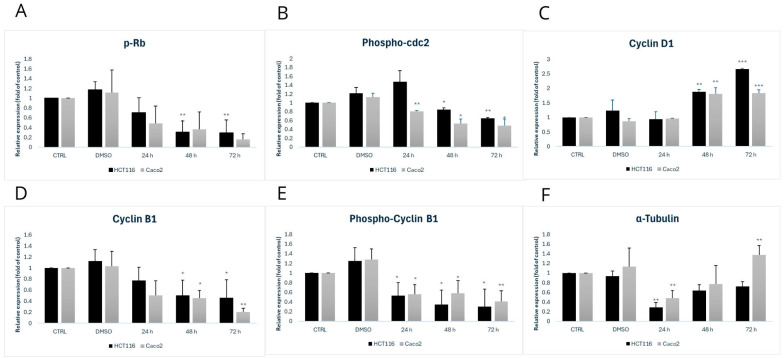
Densitometric analysis of phospho-Rb (**A**), phospho-cdc2 (**B**), Cyclin D1 (**C**), Cyclin B1 (**D**), phospho-Cyclin B1 (**E**) and α-Tubulin (**F**). Statistical significance: * *p* < 0.05, ** *p* < 0.01, *** *p* < 0.001 vs. control.

Cyclin D1 is a major protein that plays an important role in cell cycle regulation, specifically in the transition from the G1 phase to the S phase. It binds to CDK4 and CDK6. The downstream CycD1/CDK4/6 complex plays a key role in cell cycle regulation, and its control is essential for proper cell cycle progression. Deregulation of Cyclin D1 can lead to uncontrolled cell proliferation and is often associated with cancer. In the HCT116 and Caco2 cell lines, we observed an increase in this protein (48 and 72 h), as shown in Figure 2 and Figure 3C.

Cyclin B1 is involved in the regulation of mitosis, activation of the mitotic complex, chromosome condensation, nuclear membrane breakdown, and cell cycle control, ensuring that the cell does not progress to the next phase of division, thus preventing errors during cell division. In the case of the phosphorylated form of Cyclin B1, we observed a significant decrease in protein levels at 24 h, 48 h, and 72 h. We also observed a decrease in Cyclin B1 itself (Figure 2 and Figure 3D).

The phosphorylated form of cyclin-dependent kinase 1 (p-CDC2), together with Cyclin B1, forms a complex that is critical for entry into mitosis. Furthermore, p-CDC2 is involved in nuclear membrane regulation, chromosome condensation, mitotic spindle formation, and regulation of Cyclin B1 degradation. During late mitosis, p-CDC2 assists in marking Cyclin B1 for degradation Via the ubiquitin-proteasome system, leading to the inactivation of the CDC2 complex and allowing for the termination of mitosis and transition to the G1 phase. In the HCT116 cell line, we observed an increase at 24 h, followed by a decrease at 48 h and 72 h. In Caco2 cells, we observed a decrease at 24 h, 48 h, and 72 h (Figure 2 and Figure 3B).

α-Tubulin forms the basic building unit of microtubules, which are part of the cytoskeleton. Its main functions include the formation of microtubules together with β-tubulin, maintenance of cell shape, intracellular transport of vesicles and organelles, and chromosome segregation during mitosis. In HCT116 and Caco2 cell lines, we observed a significant decrease at 24 h (Figure 2 and Figure 3F).

Phosphorylated retinoblastoma protein (p-Rb) plays a key role in cell cycle regulation during the transition from G1 to S phase. Upon phosphorylation of Rb by cyclin-dependent kinases, the transcription factor E2F undergoes a conformational change and is released, which activates gene expression for DNA synthesis. In this way, p-Rb secures the cell’s entry into the DNA replication and cell proliferation phase. For p-Rb, in the HCT116 cell line, we observed a decrease in expression at all time points with the highest significance * *p* < 0.05 at 48 and 72 h. In Caco2 cells, we observed a similar trend in p-Rb expression (Figure 2 and Figure 3A).

### 3.3. Apoptosis Detection

#### 3.3.1. Impact of MB-653 on MMP

**Figure 4:**
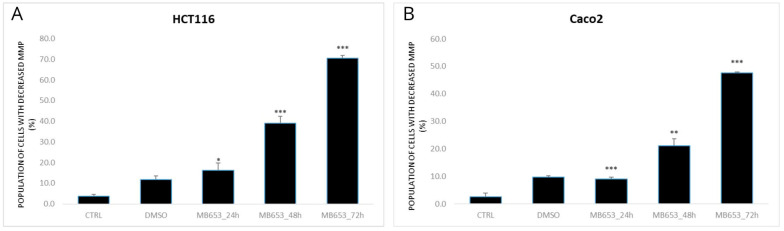
Effect of MB-653 on the loss of mitochondrial membrane potential in HCT116 (**A**) and Caco 2 (**B**) cells after 24, 48, and 72 h exposure. Data were obtained from 3 independent acquisitions and are presented as the mean ± standard deviation. Statistical significance: * *p* < 0.05, ** *p* < 0.01, *** *p* < 0.001 vs. control.

The mitochondrial membrane potential (MMP) is an electrical gradient across the inner mitochondrial membrane. It is important for ATP formation through oxidative phosphorylation and cellular respiration. Additionally, it facilitates the transport of metabolites and ions across the inner mitochondrial membrane and plays a role in the regulation of apoptosis, as a decrease in MMP may indicate the onset of apoptosis due to its association with the release of proapoptotic factors. In our measurements, we observed an increase in the population of cells with decreased MMP after incubation with MB-653 in both HCT116 and Caco2 cell lines at 24, 48, and 72 h, as shown in Figure 4.

#### 3.3.2. Annexin V–Alexa Fluor^®^ 647/PI Staining

In the process of apoptosis, we can observe changes in the structure of the cell membrane, which are manifested by the externalization of phosphatidylserine, a key component of the cell membrane. For staining, we used Annexin V, which specifically interacts with phosphatidylserine on the cell membrane surface, and PI, which serves as a DNA intercalator capable of diffusing across membranes with compromised integrity. This double staining method is used to distinguish between live (An−/PI−), early apoptotic (An+/PI−), late apoptotic (An+/PI+), and dead (An−/PI+) cells (Supplement Figure S2). As shown in Table 4, our analysis demonstrated that MB-653 significantly reduced the population of viable HCT116 cells after 48 h and 72 h and Caco2 cells after 72 h, as shown in Table 5 and Table 6. For early and late apoptotic cells, we observed a significant increase in HCT116 cells at 48 h and 72 h and in Caco2 cells at 72 h. Additionally, we noted an increase in dead cells at 48 h and 72 h in the HCT116 cell line, with a similar trend observed in the Caco2 cell line.

#### 3.3.3. Activity of Caspase 3/7

**Figure 5:**
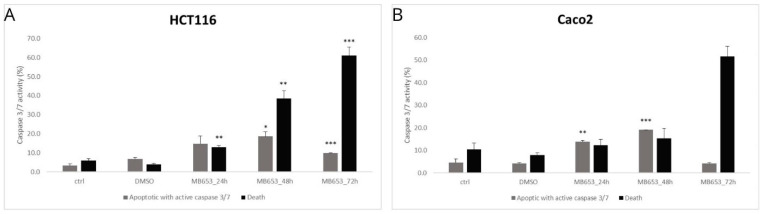
Flow cytometric analysis of activated caspase-3/7 after 24, 48, and 72 h of MB-653 treatment in HCT116 (**A**) and Caco2 (**B**) cells. Data were obtained from 3 independent acquisitions and are presented as the mean ± standard deviation. Statistical significance: * *p* < 0.05, ** *p* < 0.01, *** *p* < 0.001 vs. control.

After mitochondrial damage and cytochrome c release, caspases can initiate apoptosis. These cysteine–aspartate proteases are activated during the initial or execution phase of apoptosis. In this experiment, we assessed the proportion of live, apoptotic, and dead cells Via caspase-3/7 activity along with staining of dead cells with SYTOX™ AADvanced™. Figure 5 shows caspase-3/7 activation in HCT116 and Caco2.

Compound MB-653 induces increased apoptosis Via caspase 3/7 activation in HCT116 cells, with the most pronounced increase observed after 48 h (significance ** *p* < 0.01). Subsequently, there is a decrease in apoptotic cells after 72 h, indicating that many cells have died, as evidenced by the significant increase in total cell death (* *p* < 0.001). We further observed that MB-653 induces increased apoptosis with active caspase 3/7 in Caco2 cells, with the most pronounced increase occurring after 48 h (significance ** *p* < 0.01). Subsequently, there is a slight decrease in apoptotic cells after 72 h, suggesting that many cells may have transitioned to a state of complete death, as indicated by the increase in total cell death. These findings confirm that MB-653 has a strong proapoptotic effect on the Caco2 cell line, demonstrating a time-dependent profile. Dot blot diagrams from the flow cytometer show caspase 3/7 activity Via a substrate assay (Supplement Figure S3. The quadrants are defined as follows: Q4 = Live (no activity), Q3 = Early Apoptotic with caspase activity, Q2 = Late Apoptotic (partially disrupted membrane permeability), and Q1 = Death (with disrupted membrane permeability and no caspase activity). In Figure 5, we present the average data from Q3, which clearly represents apoptotic cells with induced caspase 3/7 activity, and a summary from Q2 + Q1, which represents dead cells with disrupted membrane permeability. Although Q2 (Late apoptotic cell population) also shows partial caspase 3/7 activity, the signal is reduced due to increased membrane permeability, causing fluorescence substrate to flow out of the cells.

#### 3.3.4. AO/PI Fluorescent Staining

**Figure 6:**
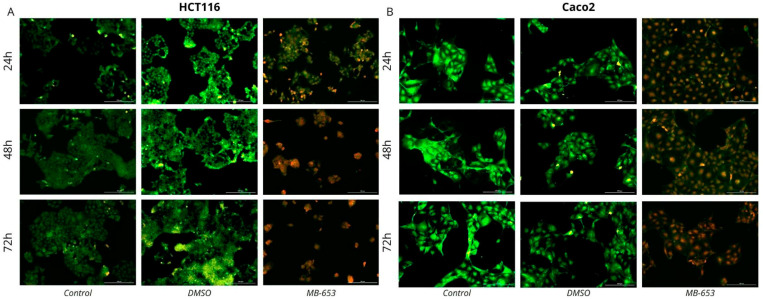
Fluorescence microscopic analysis of MB-653 induced apoptosis in HCT116 (**A**) and Caco2 (**B**) cell lines after 24, 48, and 72 h of treatment (10 µmol/L for HCT116 and Caco2) using AO/PI staining. Green shows live cells, yellow shows cells in the initial phase of apoptosis, orange shows cells in the advanced stage of apoptosis, and red indicates dead or necrotic cells. This image is a representative example from three separate experiments. Magnification is set at 100×.

Using AO/PI staining, we examined changes in membrane permeabilization that could indicate ongoing cell damage and cell death after incubation with MB-653. AO can diffuse into cells with intact membrane integrity, while PI can penetrate cells with compromised membrane integrity. This method allows cell populations to be divided into live (green), apoptotic (yellow/orange), and dead cells (red). Our results show a significant decrease in the number of live cells after incubation with MB-653 at 24, 48, and 72 h and a significant increase in the number of potentially apoptotic and dead cells with partially or completely disrupted membranes (Figure 6).

### 3.4. Effect of MB-653 on Proliferation-Regulating Proteins

**Figure 7:**
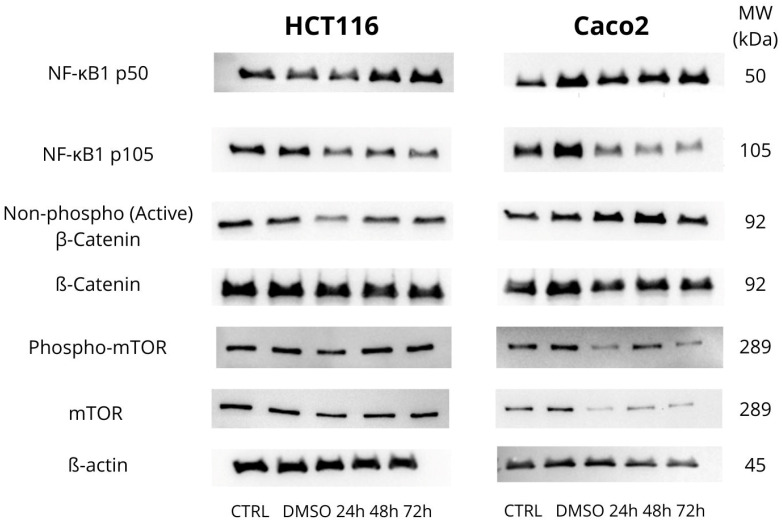
Western blot analysis of the effect on proliferation-associated proteins affected by MB-653 in HCT116 and Caco2 cells after 24, 48, and 72 h incubation. Representative figure. Data were obtained from 3 independent acquisitions.

**Figure 8:**
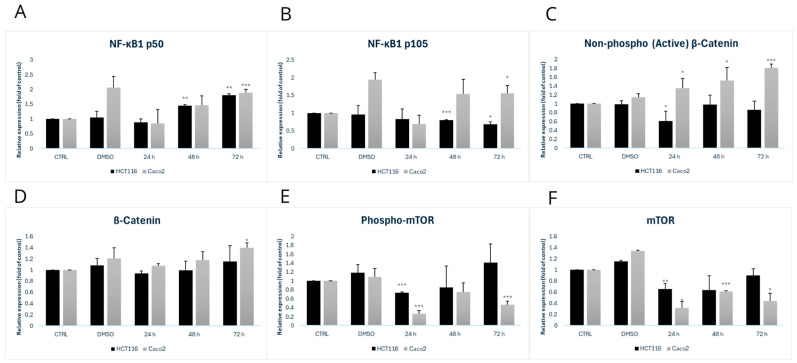
Densitometric analysis of NF-κ1 B p50 (**A**), NF-κ1 B p105 (**B**), Non-phospho (Active) β-Catenin (**C**), ß-Catenin (**D**), phospho-mTOR (**E**) and mTOR (**F**). Data were obtained from 3 independent acquisitions and are presented as the mean ± standard deviation. Statistical significance: * *p* < 0.05, ** *p* < 0.01, *** *p* < 0.001 vs. control.

Among the proteins studied, we focused on NF-κB1 p105-50—Figure 7 and Figure 8A,B, which plays a crucial role in regulating the immune response and protecting cells from apoptosis under stress conditions. NF-κB1 p105 serves as a precursor protein, while NF-κB1 p50 is the active form. In the HCT116 cell line, we observed a gradual increase in NF-κB1 p50 expression at 48 h and 72 h, with both time points showing a significant increase, marked by significance levels of ** *p* < 0.01. Concurrently, there was a notable decrease in NF-κB1 p105 expression at 48 h and 72 h, with significance levels of * *p* < 0.05 at 48 h and ** *p* < 0.01 at 72 h, respectively. In Caco2 cells, we noted an increase in NF-κB1 p50 expression at 48 h and 72 h, with the highest significance reached at *** *p* < 0.001 at 72 h, accompanied by an increase in NF-κB1 p105 at both 48 h and 72 h relative to control.

The Wnt/β-catenin signalling pathway plays an important role in colorectal cancer (CRC) and is one of the most prominent signalling pathways associated with this disease. Key aspects of this pathway include the modification and degradation of β-catenin, a major effector molecule in Wnt signalling, which is linked to the development and progression of CRC. We focused on the active form of β-catenin – Figure 7 and Figure 8C and observed a decrease in its expression at 24 h with a significance of * *p* < 0.05. In the Caco2 cell line, there was an increase in active β-catenin at all time points, with the highest significance of *p* < 0.001 at 72 h. For β-catenin itself—Figure 7 and Figure 8D, we observed no significant changes in the HCT116 cell line and a gradual increase in β-catenin with the highest significance of * *p* < 0.05 at 72 h in the Caco2 cells.

The mTOR protein—Figure 7 and Figure 8E, which is involved in regulating cell growth, plays a crucial role in proliferation. Dysregulation of the mTOR pathway is often associated with various malignancies, as increased mTOR activity promotes uncontrolled cell growth. In our experiment, we examined both mTOR and the active form of phospho-mTOR. For phospho-mTOR, we observed a decrease in its expression, with the significance of *** *p* < 0.001 at 24 h in the HCT116 cell line. In the Caco2 cells, we observed a consistent decrease in phospho-mTOR expression at all time points, with significance levels of *** *p* < 0.001 at 24 h and 72 h.

### 3.5. Effect of MB-653 on Proteins Involved in Regulating Invasiveness and Metastasis

**Figure 9:**
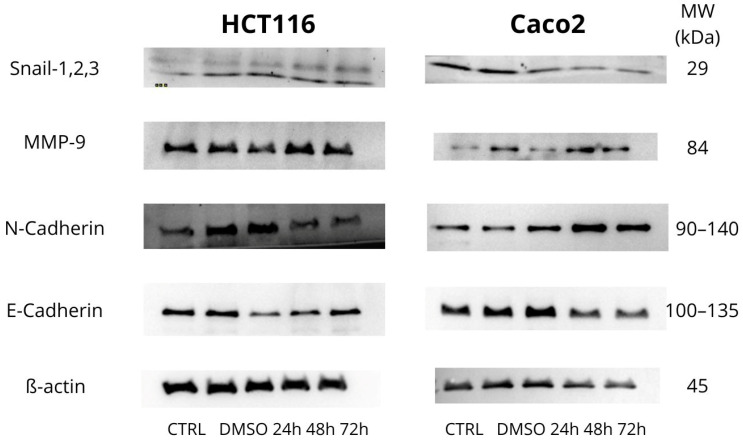
Western blot analysis of proteins responsible for invasiveness and metastasis affected by MB-653 in HCT116 and Caco2 cells after 24, 48, and 72 h incubation. Representative figure. Data were obtained from 3 independent acquisitions.

**Figure 10:**
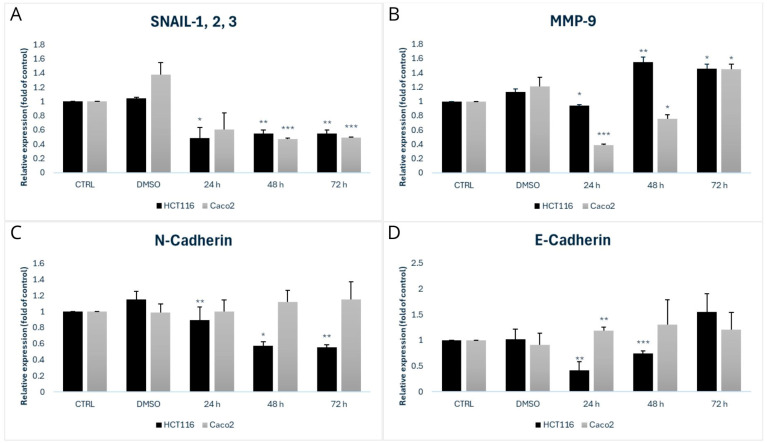
Densitometric analysis of Snail—1,2,3 (**A**), MMP-9 (**B**), N-Cadherin (**C**) and E-Cadherin (**D**). Data were obtained from 3 independent acquisitions and are presented as the mean ± standard deviation. Statistical significance: * *p* < 0.05, ** *p* < 0.01, *** *p* < 0.001 vs. control.

We focused on the detection of MMP-9, Snail 1/2/3, N-Cadherin, and E-Cadherin proteins as part of studying invasiveness and metastasis, as shown in Figure 9 and Figure 10.

Snail 1/2/3 are part of a family of transcription factors that play a critical role in processes such as epithelial–mesenchymal transition (EMT), cell migration, invasiveness, and resistance to apoptosis. These factors are primarily recognized for their involvement in regulating EMT, a pivotal process in the development of metastases. Our analysis of the HCT116 cell line revealed a significant decrease in expression at 24 h (* *p* < 0.05) and further significant reductions at 48 and 72 h (** *p* < 0.01). For the Caco2 cell line, we observed a decrease at 24 h, with more significant decreases at 48 and 72 h (*** *p* < 0.001)—Figure 9 and Figure 10A.

Matrix Metalloproteinase 9 (MMP-9) belongs to the family of matrix proteinases that degrade various components of the extracellular matrix, such as type IV and V collagen. MMP-9 plays roles in inflammatory responses, wound healing, and angiogenesis, as well as in the pathogenesis of cancer, metastasis formation, arthritis, cardiovascular diseases, and more. In the case of the HCT116 cell line, we observed a significant decrease in MMP-9 expression (* *p* < 0.05) 24 h after exposure, while an increase was observed at 48 and 72 h (** *p* < 0.01 and * *p* < 0.05, respectively), indicating dynamic changes in MMP-9 expression over time. For the Caco2 cell line, a significant decrease in expression (*** *p* < 0.001) was noted at 24 h, followed by an increase at 48 and 72 h (* *p* < 0.05)—Figure 9 and Figure 10B.

We then focused on N-Cadherin, which is a transmembrane protein responsible for adhesion between neighbouring cells. The main roles of N-Cadherin include cell migration, maintenance of tissue integrity, and morphogenesis, while in pathological conditions, it is primarily involved in tumour progression and metastasis. For the HCT116 cell line, we observed a significant decrease in expression at 48 h (* *p* < 0.05) and 72 h (** *p* < 0.01), whereas in Caco2 cells, we observed an increase at 24, 48, and 72 h—Figure 9 and Figure 10C.

E-Cadherin is a transmembrane glycoprotein that belongs to the cadherin family. Dysfunction in E-Cadherin is commonly associated with tumour progression and metastasis. A reduced expression of this protein is characteristic of cells that have undergone epithelial–mesenchymal transition (EMT), a process by which cells acquire invasive and migratory properties. For the HCT116 cell line, we observed a statistically significant decrease in E-Cadherin expression at 24 h (** *p* < 0.01). At 48 h, there was a further significant decrease (*** *p* < 0.001). Conversely, in the Caco2 cell line, we observed an increase in expression at 24 h (** *p* < 0.01), which continued at 48 and 72 h—Figure 9 and Figure 10D.

### 3.6. Substance MB-653 Is Safety for Topical and Parenteral Applications

**Figure 11:**
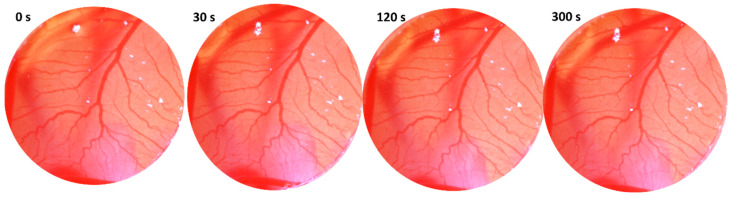
Substance MB-653 in concentration 50 µmol/L has no irritant activity at 30 s, 120 s, and 300 s after application. Data were obtained from 3 independent acquisitions.

The irritant potential was determined in a chicken model that is reliable for animal and, in addition, human responses. The CAM is characterized by its high vascularization, making it analogous to various mucosal tissues, including ocular, nasal, vaginal, and oral. Three adverse effects are classified—hemorrhage, coagulation, and lysis of blood vessels. In our case, MB-653 at concentrations (10, 25, and 50 µmol/L) did not show any of these effects on blood vessels. In addition, neither vasodilatation nor vasoconstriction was observed (Figure 11). Based on these findings, we can conclude that our agents have no negative effect on blood vessels or mucosa and can be safely used for topical and parenteral applications.

### 3.7. The Substance MB-653 Does Not Affect the Process of Angiogenesis

**Figure 12:**
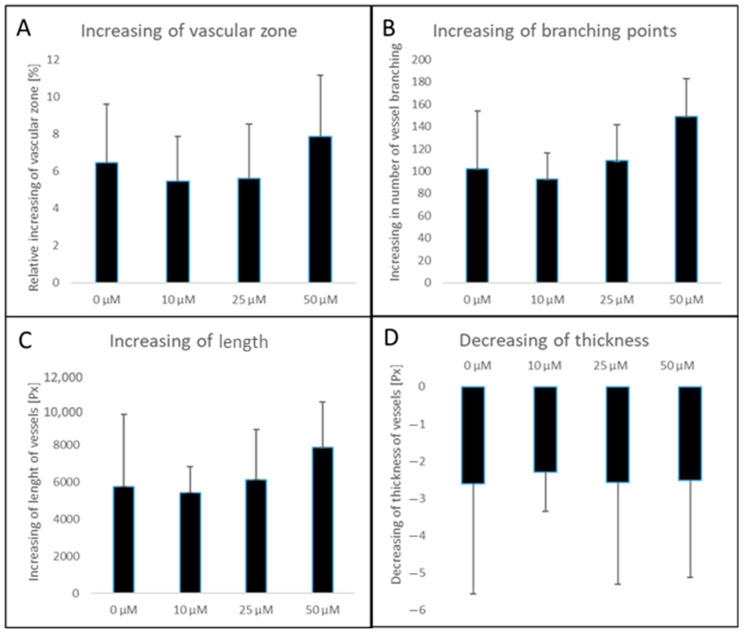
Measured angiogenesis parameters—vascular zone (**A**), number of branching points (**B**), total length (**C**), average thickness of vessels (**D**) after application of 10, 25, and 50 μM MB-653 according to software IKOSA. Px—pixels. Data were obtained from 3 independent acquisitions.

The CAM of the chicken embryo serves as an effective model for studying angiogenesis due to its highly vascularized structure. Artificial intelligence used by the IKOSA platform was used to evaluate angiogenesis by measuring four different parameters—vascular area (Figure 12A), number of branching points (Figure 12B), total length (Figure 12C), and average thickness (Figure 12D). All measured parameters used to determine angiogenesis were found to be similar in all samples and the control. Notably, the substance MB-653 did not exert any significant positive or negative effects on the angiogenic process.

## 4. Discussion

Colorectal cancer is among the most prevalent malignancies, exhibiting a significant fatality rate [15]. The observation that heightened intake of cruciferous vegetables, abundant in indole phytoalexins, may diminish the risk of this specific cancer type and other gastrointestinal cancers [16] initiates further investigations in this field.

We and other scholars previously detected advantageous activity of indole phytoalexins or their synthetic derivatives towards different cancer cell lines via intracellular glutathione (GSH) content reduction [12], ROS production [17], mitochondrial-mediated apoptosis induction [13], autophagy regulation [18], or synergism with conventional chemotherapeutic agents [19].

The present study aimed to evaluate the mode of cytotoxic, proapoptotic, and anti-invasive effects of the synthetic indole phytoalexin MB-653 in human colorectal cancer cell lines.

Our results showed a concentration-dependent antiproliferative effect of compound MB-653 in both colorectal cancer cell lines with IC_50_ values of 5.8 ± 0.3 µmol/L and 6.1 ± 2.1 µmol/L for the HCT116 and Caco2 cell lines, respectively. We also detected slightly lower sensitivity of non-cancer cells to the studied compound, which is in agreement with studies having described earlier their selective cytotoxicity [20,21]. Dysregulated and aggressive proliferation and rapid growth of the tumour cells are one of the major characteristics of carcinogenesis. Cancer cells develop the ability to grow uncontrollably, and they generate their growth signals and become insensitive to antigrowth signals; that is why modulating uncontrolled proliferation represents an attractive strategy in fighting cancer [22].

To verify the assumption that the antiproliferative activity of substance MB-653 may be associated with cell cycle arrest, in the following phase, we performed cell cycle analysis using flow cytometry. Cell cycle control is one of the major regulatory mechanisms of cell growth, and abnormal regulation of the cell cycle is a notable characteristic of cancer cells [23]. After MB-653 treatment, the Caco2 and HCT 116 cell cycle was inhibited in the G2/M phase, with significance after 72 h of incubation. These results are supported by other published studies using several other types of human colon cancer cells, including Caco2 and HT-29 [17,24]. It is worth noting that a key regulator of the G2/M transition of the cell cycle is a complex of Cdk1 (=cdc2)/Cdk2 and a B-type cyclin [25]. In the present study, the protein expression levels of cyclin B (both total and phospho) and p-Rb (tumour-suppressor) were downregulated in MB-653 treated colon cancer cells.

The phosphorylation of cyclin B1 facilitates its translocation and concentration in the nucleus, so initiating mitosis. In certain cancer types, this cyclin may be overexpressed, correlating with a poor prognosis [26]. The phosphorylation of cdc2 at Tyr15 diminishes its activity [27], hence inhibiting cell cycle progression and the transition of cells into mitosis. Unexpectedly, exposure of both HCT116 and Caco2 cells to substance MB-653 resulted in a considerable, statistically significant, decrease in levels of phosphorylated cdc2 (48 h and 72 h of incubation). Although in most experimental works, G2/M phase cell cycle arrest of cancer cells was accompanied by increased cdc2 phosphorylation [28,29], this phenomenon is not absolutely unique, as reduced cdc2 phosphorylation and arrest of cell cycle at G2/M phase has also been described previously in different cancer cell lines by naturally based compounds [30,31]. Additionally, because phosphorylation of cyclin B1 is important for cyclin-B1/cdc2 kinase activation [32], we suggest that treatment of MB-653 triggers a dose-dependent accumulation of G2/M phase colon cancer cells through the dysfunction of the cyclin B1/CDC2 complex. Furthermore, Western blot analysis showed that α-tubulin protein levels decreased in both HCT116 and Caco2 cells. Although we do not have direct evidence for such a claim, we hypothesize that G2/M arrest induced by MB-653 might stem, in part, from its microtubule targeting activity, as well. The indole structure is linked to interacting with tubulins, a significant target in anticancer therapy. The utilization of indole compounds to interact with tubulins is a prominent focus in anticancer therapeutic research [33]. To the best of our knowledge, this is the first study to detect alterations in tubulin function /expression and G2/M phase halt in colorectal cancer cells by indole phytoalexins.

In addition to the role of tubulins in cell proliferation, their disintegration may also lead directly to apoptosis [34]. The critical hallmark features for cancer initiation and progression are primarily assigned to the defect in the apoptotic signalling pathways [35]. Indole phytoalexins are considered to be potent triggers of apoptosis [36,37,38], which was confirmed also in our study. However, not all mechanisms proposed to explain such proapoptotic action of indole phytoalexins were fully described, and they may differ to some extent.

Data from experimental studies point out the potential of natural compounds targeting mitochondria for CRC therapy [39]. This can be accomplished through various mechanisms, including the inhibition of the mitochondrial respiratory chain, the increase in reactive oxygen species (ROS), the disruption of mitochondrial membrane potential (∆ψm), the release of proapoptotic factors, the modulation of the *Bcl*-2 protein family to facilitate the release of cytochrome c, the initiation of apoptotic vesicle activity through the activation of the caspase protein family, and the selective targeting of mitochondrial division [40,41]. In our analyses, the proapoptotic activity of the studied phytoalexin was confirmed by evaluation of the 3 and 7 caspases and mitochondrial dysfunction as evidenced by the increase in several cells with dissipated ∆ψm. Moreover, in our study, we observed a time-dependent significant increase in the number of apoptotic colon cancer cells, as evidenced by annexin V/PI and AO/PI staining as well. These results favour the intrinsic apoptotic pathway as a primary mechanism of cell death induced by MB-653 in HCT116 and Caco2 cells.

Another important finding, the treatment of HCT116 and Caco2 cells with MB-653 resulted in the modulation of several signalling pathways associated with different cellular activities, such as cell survival, proliferation, tumour invasion, metastasis, and EMT (epithelial–mesenchymal transition). This fact confirms available data from the literature that the effects of indole phytoalexins appear to be multitargeted.

Among those pathways, the special mention requires the Wnt/β-catenin signalling cascade as one of the most important developmental pathways in CRC oncogenesis [42]. Worth mentioning the level of unphosphorylated beta-catenin is a key factor in the regulation of the Wnt/β-catenin signalling pathway. In many types of cancer, for example, colorectal or hepatocellular, this pathway is hyperactivated, leading to the accumulation of unphosphorylated beta-catenin in the cell nucleus. This stimulates genes promoting cell proliferation and resistance to apoptosis, thereby promoting tumour growth [43]. In such cases, it is therefore desirable to reduce the level of unphosphorylated beta-catenin in order to limit the growth of tumour cells and their ability to resist apoptosis [44]. The proapoptotic and antiproliferative activity of MB-653 was associated with suppression of non-phospho (p)-β-catenin expression exclusively in HCT116 cells. Ye et al. (2019) [45] recently reported that 4b-hydroxywithanolide E (4b-HWE), a natural withanolide from *Physalis peruviana*, increases the level of phosphorylated b-catenin and decreases the levels of active nonphosphorylated form and total β-catenin in colon cancer HCT116 cells. Interestingly, in Caco2 cells we observed an opposite trend in unphosphorylated beta-catenin levels changes under the treatment of MB-653. Important to note that stimulation of this pathway is considered for the immunotherapy of certain tumours as well as to promote the differentiation of cancer stem cells into less aggressive forms more sensitive to treatment [46,47].

In addition to the aforementioned role of β-catenin, it also has been documented its crucial role in cell–cell adhesion by binding with E-cadherin, thus giving rise to the E-cadherin/catenin complex [48]. This complex has been acknowledged as essential for cell adhesion and tissue homeostasis. The loss of cell adhesion is seen as a critical step in the cascade that leads to cancer spread. The diminished expression of E-cadherin, an epithelial marker, has been documented in multiple malignancies, correlating with tumour growth, metastasis, and reduced overall survival [49]. Conversely, elevated levels of N-cadherin and vimentin, which are mesenchymal markers, facilitate metastasis and invasiveness [50]. In more detail, an upregulation of N-cadherin followed by the downregulation of E-cadherin is a common indicator of a process known as EMT, during which epithelial cells lose their intracellular adhesion and cell polarity to gain a motile mesenchymal phenotype [51]. EMT is a multistep reversible process and an initial step of the metastatic cascade [52].

Our results indicate that MB-653 exerts EMT-modulating activity. After MB-653 treatment we observed an increase in E-cadherin protein levels in Caco2 cells, whilst no significant changes were observed in N-cadherin protein expression. In HCT116 cells, we detected a significant decrease in N-cadherin protein and, surprisingly, a significant decrease in E-cadherin protein level. Although the prevailing belief is that induction of EMT and loss of E-cadherin are a prerequisite for progression to metastatic disease [53], E-cadherin loss does not always correlate with EMT. Reducing the level of E-cadherin in the treatment of tumours is, in most cases, considered an undesirable effect because it is related to an increase in the invasiveness and metastatic potential of tumour cells. However, under certain specific circumstances and with some therapeutic strategies, E-cadherin downregulation may be considered a desirable effect, especially if it contributes to a higher treatment efficacy or to a change in cell phenotype that is therapeutically beneficial. For example, some studies show that reducing E-cadherin expression has led to weakened cell contacts and lower tumour tissue integrity, theoretically leading to improving drug penetration into tumour tissue [54,55].

The research by Yang et al. in 2019 [56] investigated the effects of brassinin, natural indole phytoalexin, on the EMT-related cell signalling cascade. Their findings demonstrated that brassinin downregulates various mesenchymal markers while upregulating epithelial markers. It effectively suppressed the expression of TGF-β-induced fibronectin, vimentin, N-cadherin, the matrix metalloproteinases (MMP-9 and MMP-2), Twist, and Snail while increasing TGF-β-induced occludin and N-cadherin levels. Modulating these EMT-related factors leads to decreased proliferation and invasion in lung carcinoma cells, specifically A549 and H1299 cells.

Next, our results indicate that MB-653 inhibits the invasivity of colon cancer cells by decreasing Snail (Caco2 and HCT116) and MMP-9 (Caco2). Twist and Snail are considered the major transcription factors modulating EMT in various cancer types by repressing E-cadherin transcription [57]. Matrix metalloproteinases (MMPs) are a substantial family of zinc-dependent neutral endopeptidases and have been demonstrated to be downstream targets of the Wnt signalling pathway [58]. Prior research has shown that MMP2, MMP3, and MMP9 can modulate the breakdown of nearly all extracellular matrix constituents, hence enhancing tumour motility, invasion, and metastasis [59]. As far as we know, this is the first research from the point of view of colorectal carcinoma EMT modulation by the group of indole phytoalexins.

For a long time, NF-κB was presumed to only regulate cancer cell proliferation and resistance to apoptosis. Additional roles of NF-κB in cancer biology, such as in tissue invasion, migration, and metastasis, have only been discovered recently. Importantly, NF-κB is involved in inflammation-induced cancer development and progression [60] and has been identified as a significant regulator of EMT in several cell types [61,62,63]. By using Western blot analysis, we observed a modulation in protein levels of NF-κB p105/50 in MB-653-treated colorectal cancer cell lines of both types, suggesting the NF-κB pathway role in EMT-regulating activity of the tested compound. The levels of the p50 polypeptide (in its active form) increased in a time- and concentration-dependent manner. NF-kB generally exists as a heterodimer of the p50 and p65 polypeptides. Although generally acting as an anti-apoptotic regulator, under specific conditions, NF-κB may also exert apoptosis-inducing properties [64,65]. In the absence of p65, p50 homodimers may act as repressors of certain antiapoptotic genes (e.g., genes from the *Bcl*-2 family), thus making cancer cells more vulnerable and prone to apoptosis [66,67]. However, because the protein levels of the p65 cofactor were not analyzed in our study, it is not known to what extent the change in p65 levels could have contributed to the complex action of the substance.

The safety of the application was evaluated using the HET-CAM assay, a method recommended by the National Institutes of Health. This assay utilizes the highly vascularized CAM of the chicken embryo, which serves as an excellent model for testing a variety of products, including cosmetic [67], vaccine adjuvants [68], dental products [69,70], medical gas plasma [71], drugs [72,73], potential pharmaceuticals [74,75] or nanoparticles [76].

The use of chicken embryos as an alternative for in vivo safety assessment is particularly advantageous due to their lack of pain perception until day 15 post-fertilization. To evaluate irritant potential, we monitored hemorrhage, coagulation, and lysis of blood vessels over time. The non-irritant potential of the substances indicates their suitability for application to vaginal mucosa [67], oral mucosa [77], nasal mucosa, or for ophthalmic use [73]. In our study, no negative effects on the vascular area were observed following the application of various concentrations of the tested substance MB-653 (10, 25, and 50 µmol/L). Furthermore, it has been demonstrated that the substance can be safely delivered Via the nasal route without irritation [78]. Except for topical application, this method also determines the safety of parenteral applications, such as subcutaneous, intramuscular, and intravenous administration [68]. The application of substance MB-653 is not expected to induce an inflammatory response that results in pain or tissue damage [68]. In conclusion, our study demonstrated that the derivative exhibited no irritant potential, as assessed by the HET-CAM assay, indicating its suitability for therapeutic use in these contexts. According to our knowledge, the safety of indole phytoalexins using this method has not yet been evaluated.

The effect on angiogenesis was determined by four different parameters. The vascular surface area is the surface of all the vessels in the area under study and increases during angiogenesis due to the formation of new vessel sprouts. The number of branching points is the main parameter measured to assess angiogenesis. Each new vascular branch develops from pre-existing vessels by intussusception or sprouting [79]. The total length of the vascular network increases during angiogenesis due to the formation of new vessels and the elongation of pre-existing vessels [80]. The diameter of the new vessels is smaller than the diameter of the pre-existing vessels. This leads to a decrease in the average thickness of the vascular network during angiogenesis [81]. Different concentrations of MB-653 (10, 25, and 50 µmol/L) and saline (0.9% NaCl) had similar increases in vessel branching, vessel area and total branching. The mean thicknesses decreased at the same level after 72 h in all these cases. According to these four methods, we can conclude that the newly synthesized sub-stance MB-653 in the three concentrations mentioned above does not affect angiogenesis positively but neither negatively. Brassinin, a natural indole phytoalexin, was found to inhibit angiogenesis in an ex vivo aortic ring assay and an in vivo Matrigel plug assay. According to the authors, brassinin was identified as a potential antiangiogenic drug for future TNBC (triple-negative breast cancer) cancer treatment [82].

## 5. Conclusions

Our experiments were originally run due to a lack of data documenting the role of indole phytoalexins and their synthetic derivatives in relation to colon cancer molecular behaviour. The key results obtained in our research can be summarized in a few paragraphs.

First of all, we found a concentration and time-dependent antiproliferative effect of tested substance MB-653 on both HCT116 and Caco2 cell lines, as documented by the MTT assay. In the next phases of our research, we focused on the mechanisms of this action in more detail.

Important to note after MB-653 treatment, the Caco2 as well as HCT 116 cell cycle was inhibited in the G2/M phase, with significance after 72 h of incubation. We suggest that the mechanisms which could have been responsible for the suppression of cell cycle progression were the dysfunction of the cyclin B1/CDC2 complex and/or α-tubulin protein levels decrease.

Indole phytoalexins are considered to be potent triggers of apoptotic processes, which was also confirmed in our study. Our findings demonstrated that MB-653 induced caspase-dependent apoptosis in both colon cancer cell lines. The proapoptotic activity was evidenced by the activation of caspases 3 and 7, mitochondrial membrane potential decline, and an increased number of apoptotic cells, confirmed by annexin V/PI and AO/PI staining.

In addition, MB-653 modulated several important pathways associated with tumour progression, invasiveness, and metastasis. Among those pathways, the special mention requires the Wnt/β-catenin signalling cascade as one of the most important developmental pathways in CRC oncogenesis, whereas changes in unphosphorylated beta-catenin (active) levels were detected. EMT (epithelial–mesenchymal transition)-related cell signalling cascades represent another molecular target of MB-653 as evidenced by the deregulation of cadherins, Snail, MMP-9, and NF-κB p105/50 protein levels. To the best of our knowledge, this is the first study to evaluate such a multimodal change in colon cancer cell machinery by the indole phytoalexins at pre-clinical testing.

Last but not least, no irritant effect of the compound occurred indicating the possibility of its topical as well as parenteral (such as subcutaneous, intramuscular, and intravenous) administration. Moreover, the application of substance MB-653 is not expected to induce an inflammatory response that results in pain or tissue damage.

Collectively, our data indicate a multi-targeted action of MB-653 in colon cancer cell lines influencing key characteristics of malignant processes such as uncontrolled cell growth, defect in the apoptotic signalling pathways, as well as tumour-spread enabling hallmarks. Our results provide a rationale for further in vitro and in vivo testing of MB-653, pointing out its real potential in the colorectal cancer fight.

## Figures and Tables

**Table 1:** List of primary and secondary Western blot antibodies.

Primary Antibodies	Mr (kDa)	Origin	Manufacturer
β-Actin (8H10D10) Mouse mAb	45	Mouse	Cell Signalling Technology^®^, Danvers, MA, USA
Snail 1/2/3 Polyclonal Antibody	29	Rabbit	Bioss, Woburn, MA, USA
Phospho-cdc2 (Tyr15) (10A11)	34	Rabbit	Cell Signalling Technology^®^, Danvers, MA, USA
Cyclin D1 (E3P5S) XP^®^	36	Rabbit	Cell Signalling Technology^®^, Danvers, MA, USA
Phospho-Cyclin B1 (Ser133) (9E3)	55	Rabbit	Cell Signalling Technology^®^, Danvers, MA, USA
α Tubulin (E-19)-R	55	Rabbit	Santa Cruz Biotechnology, Inc., Dallas, TX, USA
MMP-9 (D6O3H) XP	84/92	Rabbit	Cell Signalling Technology^®^, Danvers, MA, USA
Non-phospho (Active) β-Catenin (Ser33/37/Thr41) (D13A1)	92	Rabbit	Cell Signalling Technology^®^, Danvers, MA, USA
β-Catenin	92	Rabbit	Cell Signalling Technology^®^, Danvers, MA, USA
NF-κB1 p105/50	50/105	Mouse	Santa Cruz Biotechnology, Inc.
E-Cadherin (4A2)	100–135	Mouse	Cell Signalling Technology^®^, Danvers, MA, USA
N-Cadherin (D4R1H) XP	90–140	Rabbit	Cell Signalling Technology^®^, Danvers, MA, USA
Phospho-Rb (Ser807/811) (D20B12) XP	110	Rabbit	Cell Signalling Technology^®^, Danvers, MA, USA
mTOR (7C10)	289	Rabbit	Cell Signalling Technology^®^, Danvers, MA, USA
Phospho-mTOR (Ser2448) (D9C2) XP	289	Rabbit	Cell Signalling Technology^®^, Danvers, MA, USA
**Secondary Antibodies**		**Origin**	**Manufacturer**
Anti-rabbit IgG HRP		Goat	Cell Signalling Technology, Danvers, MA, USA
Anti-mouse IgG/HRP		Goat	Dako, Glostrup, Denmark

**Table 2:** Scoring scheme for the test of irritation by HET-CAM method.

Effect	Score		
	30 s	120 s	300 s
Lysis of vessels	5	3	1
Hemorrhage	7	5	3
Coagulation	9	7	5

**Table 3:** IC_50_ (μmol/L) of MB-653 and cisplatin of HCT116, Caco2, and MCF10A cell lines after 72 h of incubation.

			Colon Cancer Cell Lines		Normal Epithelial Cell Line
			HCT116	Caco2	MCF-10A
MB-653			5.8 ± 0.3	6.1 ± 2.1	10.13 ± 6.2
Cis-Pt			9.0 ± 0.4	30.7 ± 0.8	25.9 ± 0.3
Selectivity index for MB-653			1.77	1.66	1
Selectivity index for Cis-Pt			2.88	0.84	1

**Table 4:** Cell cycle analysis of HCT116 (**A**) and Caco2 (**B**) cells after treatment with MB-653 for 24, 48, and 72 h at 10 μmol/L. Data were obtained from 3 independent acquisitions and are presented as the mean ± standard deviation. Statistical significance: * *p* < 0.05, ** *p* < 0.01, *** *p* < 0.001 vs. control.

A	sub-G0	G1	S	G2/M
Control	2.8 ± 0.8	67.0 ± 1.6	13.6 ± 2.1	16.6 ± 0.3
DMSO	1.5 ± 0.2	74.8 ± 3.2	16.1 ± 1.8	7.7 ± 1.2
24 h	11.2 ± 1.9 **	50.0 ± 5.0 *	14.8 ± 0.7	24.1 ± 4.2
48 h	35.9 ± 2.5 ***	25.8 ± 2.9 ***	7.1 ± 0.4 *	31.2 ± 2.5 **
72 h	49.2 ± 2.7 ***	13.8 ± 0.1 ***	8.4 ± 1.1 *	28.7 ± 3.3 **
**B**	**sub-G0**	**G1**	**S**	**G2/M**
Control	1.2 ± 0.4	49.7 ± 3.4	20.3 ± 1.1	28.9 ± 1.9
DMSO	1.8 ± 0.1	38.8 ± 1.4	29.6 ± 2.5	29.8 ± 3.0
24 h	1.5 ± 0.5	54.8 ± 3.9	19.5 ± 1.6	24.3 ± 4.4
48 h	2.6 ± 0.7	34.8 ± 4.0 *	16.8 ± 2.2	45.8 ± 4.7 *
72 h	5.6 ± 0.5 **	31.7 ± 1.2 **	11.1 ± 0.5 ***	51.7 ± 1.2 ***

**Table 5:** Percentage distribution of HCT116 cell populations after 24, 48, and 72 h treatment with MB-653 based on Annexin V/PI staining. Data were obtained from 3 independent acquisitions and are presented as the mean ± standard deviation. Statistical significance: * *p* < 0.05, ** *p* < 0.01, *** *p* < 0.001 vs. control.

	An−/PI−	An+/PI−	An+/PI+	An−/PI+
Control	81.7 ± 2.3	9.995 ± 2.5	3.51 ± 0.4	4.82 ± 0.8
DMSO	89.1 ± 0.7	5.7 ± 0.0	2.6 ± 0.4	2.7 ± 0.3
24 h	74.6 ± 0.5	15.25 ± 0.2 *	5.49 ± 1.4	4.655 ± 0.4
48 h	33.0 ± 0.7 ***	24.65 ± 2.0	19.75± 3.9 **	22.60 ± 3.5 *
72 h	19.6 ± 0.2 ***	35.8 ± 1.9	31.65 ± 3.0 *	12.95 ± 2.1 *

**Table 6:** Percentage distribution of Caco2 cell populations after 24, 48, and 72 h treatment with MB-653 based on Annexin V/PI staining. Data were obtained from 3 independent acquisitions and are presented as the mean ± standard deviation. Statistical significance: * *p* < 0.05, ** *p* < 0.01 vs. control.

	An−/PI−	An+/PI−	An+/PI+	An−/PI+
Control	81.1 ± 3.9	8.45 ± 2.0	3.845 ± 2.3	6.58 ± 2.2
DMSO	77.3 ± 0.8	7.8 ± 0.5	3.4 ± 0.4	11.5 ± 0.8
24 h	72.2 ± 2.1	17.74 ± 4.5	4.735 ± 2.6	5.335 ± 0.5
48 h	69.15 ± 5.5	9.27 ± 2.1	8.705 ± 3.1	12.85 ± 0.3 *
72 h	44.35 ± 1.1 **	29.65 ± 2.2 **	13.82 ± 3.2 *	12.2 ± 0.1 *

## Data Availability

The data presented in this study are available from the authors upon reasonable request.

## Supplementary Materials

The following supporting information can be downloaded at https://www.mdpi.com/article/10.3390/biom15010072/s1. Figure S1. Representative histograms of flow cytometry cell cycle analyses of HCT116 and Caco-2 cell lines after 24, 48 and 72 h of treatment with MB653; Figure S2. Representative Annexin V/PI dot plot of HCT116 and Caco-2 cells after 24, 48 and 72 h of incubation with tested compound MB653; Figure S3. Representative dot plot illustrating caspase-3/7 activation after 24, 48, and 72 h of incubation with MB-653 in HCT116 and Caco-2 cells.
