# Supplementary material for: The Proapoptotic Effect of MB-653 Is Associated with the Modulation of Metastasis and Invasiveness-Related Signalling Pathways in Human Colorectal Cancer Cells
Source: Biomolecules. 2025 Jan 6;15(1):72. doi: 10.3390/biom15010072 (PMC11762530; doi:10.3390/biom15010072)
Supplement: Supplementary file 1 [file biomolecules-15-00072-s001.zip › biomolecules-3356258-supplementary.pdf]

biomolecules

MDPI

Supplementary materials: The Proapoptotic Effect of MB-653 Is Associated with the Modulation of Metastasis and Invasiveness-Related Signalling Pathways in Human Colorectal Cancer Cells

Libor Sokoli [1,2], Peter Takáč [1,*], Mariana Budovská [3], Radka Michalková [2], Martin Kello [2], Natália Nosálová [4], Ľudmila Balážová [5], Šimon Salanci [2] and Ján Mojžiš [2,*]

1 Department of Pharmacology and Toxicology, University of Veterinary Medicine and Pharmacy, Komenského 73, 041 81 Košice, Slovakia; liborsokoli@gmail.com
2 Department of Pharmacology, Faculty of Medicine, Pavol Jozef Šafárik University, 040 01 Košice, Slovakia; radka.michalkova@upjs.sk (R.M.); martin.kello@upjs.sk (M.K.); simon.salanci@student.upjs.sk (Š.S.)
3 Department of Organic Chemistry, Institute of Chemistry, Faculty of Science, Pavol Jozef Šafárik University, 040 01 Košice, Slovakia; mariana.budovska@upjs.sk
4 Small Animal Clinic, University of Veterinary Medicine and Pharmacy, Komenského 73, 041 81 Košice, Slovakia; natalia.nosalova@uvlf.sk
5 Department of Pharmaceutical Technology, Pharmacognosy and Botany, University of Veterinary Medicine and Pharmacy in Košice, 041 81 Košice, Slovakia; ludmila.balazova@uvlf.sk
* Correspondence: peter.takac@uvlf.sk (P.T.); jan.mojzis@upjs.sk (J.M.)

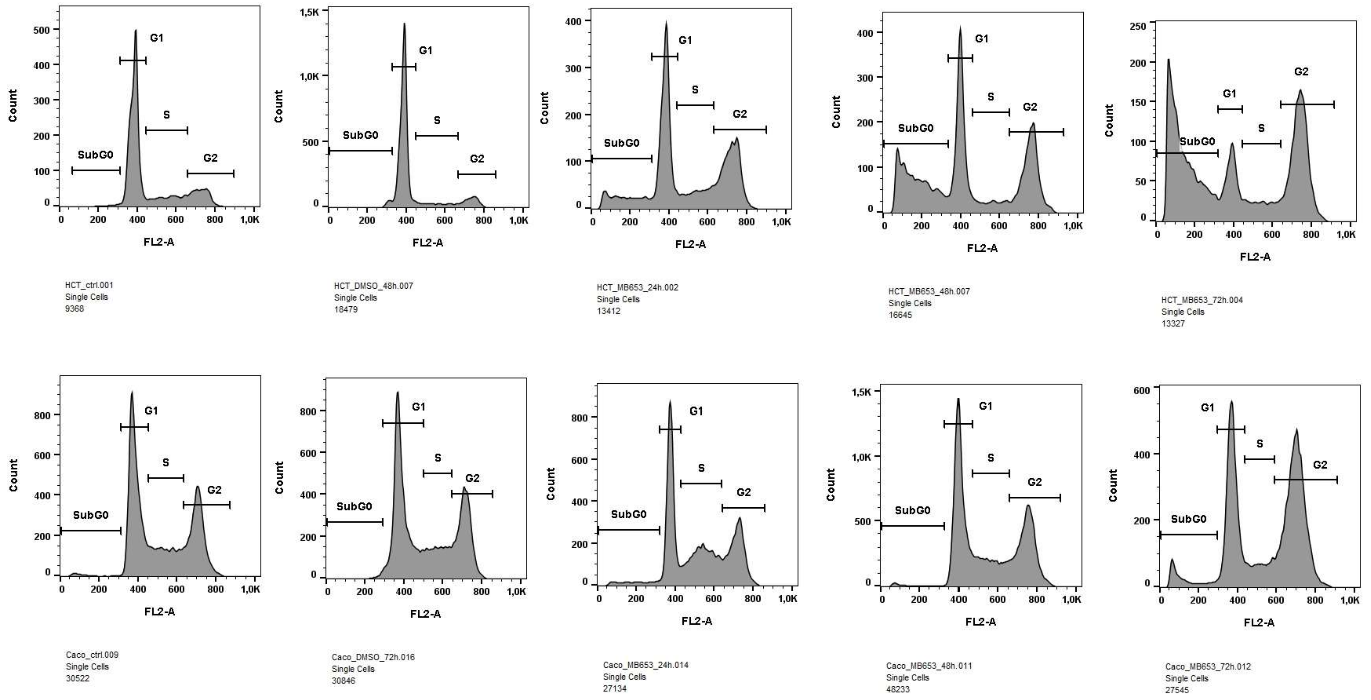

Figure S1. Representative histograms of flow cytometry cell cycle analyses of HCT116 and Caco-2 cell lines after 24, 48 and 72 h of treatment with MB653.

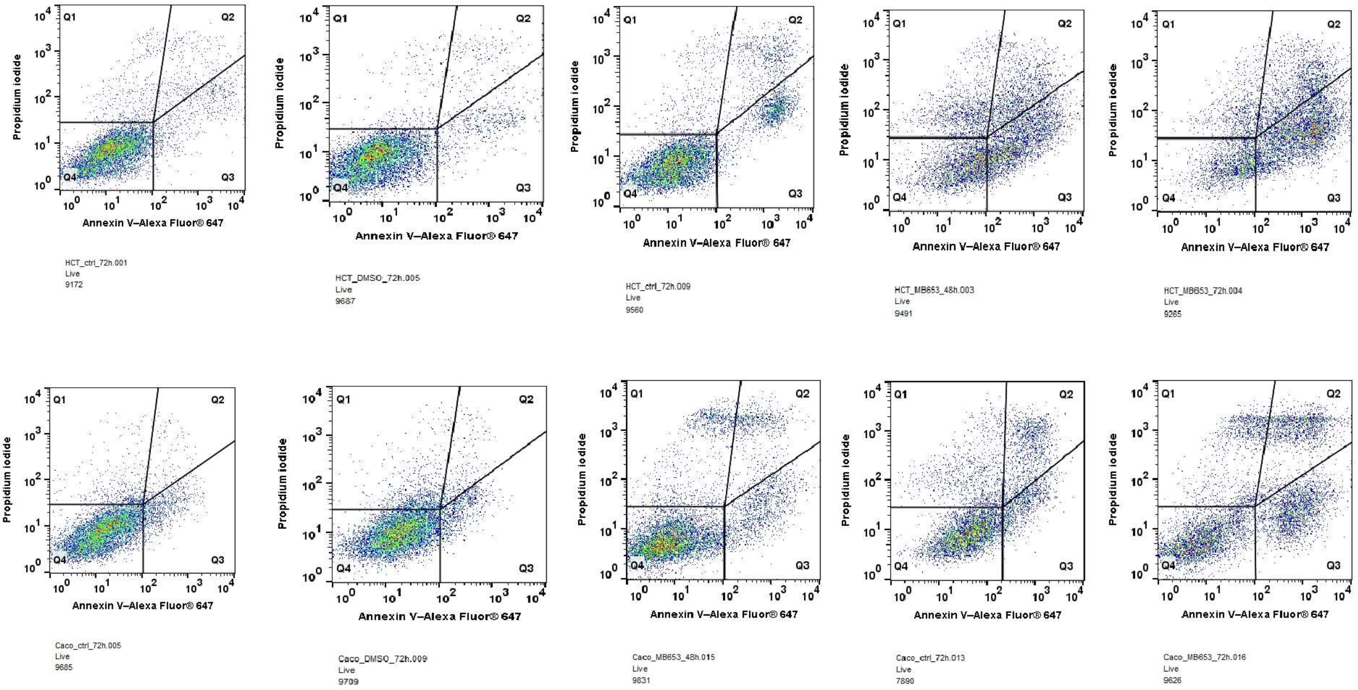

Figure S2. Representative Annexin V/PI dot plot of HCT116 and Caco-2 cells after 24, 48 and 72 h of incubation with tested compound MB653.

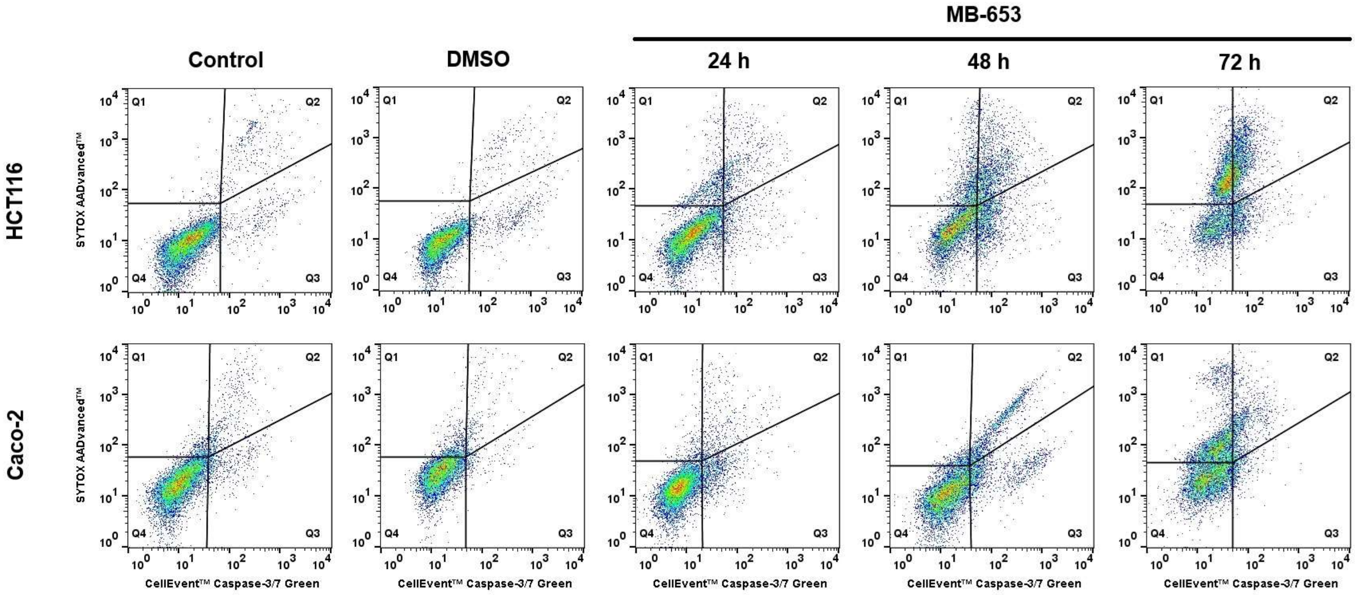

Figure S3. Representative dot plot illustrating caspase-3/7 activation after 24, 48, and 72 hours of incubation with MB-653 in HCT116 and Caco-2 cells. Quadrant Q3 indicates the apoptotic cell population with active caspase-3/7, Q1 and Q2 correspond to dead or necrotic cells, and Q4 represents live cells.
